# Impact of Length of Stay on Readmission in Hospitalized Patients

**DOI:** 10.7759/cureus.10669

**Published:** 2020-09-26

**Authors:** Jean-Sebastien Rachoin, Kara S Aplin, Snehal Gandhi, Eric Kupersmith, Elizabeth Cerceo

**Affiliations:** 1 Critical Care, Cooper University Hospital, Camden, USA; 2 Hospital Medicine, Cooper University Hospital, Camden, USA

**Keywords:** hospital readmission rate, hospitalized patients, length of stay (los)

## Abstract

Background

Readmission and length of stay (LOS) are two hospital-level metrics commonly used to assess the performance of hospitalist groups. Healthcare systems implement strategies aimed at reducing both. It is possible that tactics aimed at improving one measure in individual patients may adversely impact the other.

Objective

We sought to analyze the impact of length of stay on readmission risk in an inpatient general medical population to assess whether patients with a lower length of stays were readmitted more frequently to the hospital.

Methods

We performed a retrospective analysis of inpatient adult patients admitted to our institution between January 2016 and December 2019. We recorded demographic variables and the outcomes of LOS and 30-day readmission. We excluded patients who expired, left against medical advice, or were transferred to other hospitals. We performed both univariate and multivariate analyses.

Results

There were 91,723 patients included in the study of which 10,598 (11.6%) were readmitted. The geometric LOS for all patients was 5.37 days and was higher in readmitted patients (6.87 vs 5.18 days, respectively, p < 0.001). Patients with higher readmission rates were older, had a higher proportion of male gender, African-American ethnicity, and were more likely to have Medicare or Medicaid payors. After performing a multivariate regression analysis, we found that a high LOS was associated with a higher likelihood of readmission (P < 0.001).

Conclusion

Contrary to our initial hypothesis, we found that general medical patients with a higher LOS had a higher likelihood of being readmitted to the hospital after adjusting for other variables. It is possible that factors not captured in the current dataset may help explain both the increase in LOS and readmission risk.

## Introduction

The rate of 30-day readmissions and length of stay (LOS) are two hospital-level metrics commonly used to assess the performance of hospitalist groups. Thirty-day risk-standardized hospital-wide readmissions are publicly reported by the Centers for Medicare and Medicaid Services (CMS). The Hospital Readmissions Reduction Program (HRRP) is a Medicare value-based purchasing program that reduces payments to hospitals with readmission rates above certain thresholds. The program links payments to CMS-determined quality metrics. LOS is a common quality metric that hospital systems monitor in light of a prospective payment system. Prospective payment systems provide a fixed payment for procedures and diagnoses, regardless of days spent in the hospital [1]. As this model for reimbursement is utilized by CMS and private payers, hospitals are driven to seek a shorter length of stay.

Healthcare systems implement strategies aimed at reducing both readmissions and LOS. It is possible, however, that tactics aimed at improving one measure in individual patients may adversely impact the other. Studies in focused populations of patients hospitalized with a specific diagnosis have found associations between individual patient LOS and the risk of readmission. Prior studies have focused on particular diagnoses and found conflicting evidence regarding whether LOS impacts readmission risk (e.g., in protocol-focused care, such as for knee and hip replacement) [2-7]. In other focused populations, such as heart failure, results have shown that either longer or shorter LOS may be associated with an increased rate of readmission [8-9].

Some authors have measured the impact of interventions on LOS and readmissions. Multidisciplinary rounds, e.g., on a vascular surgery service, were found to decrease LOS but not lead to any significant effect on readmissions [10]. We have previously shown that decreasing observation LOS in a hospitalist-run clinical decision unit is not associated with an increased rate of emergency department visits or readmissions [11].

In this study, we sought to analyze the impact of LOS on readmission risk in an inpatient general medical population to see whether patients with a lower LOS were readmitted more frequently to the hospital.

## Materials and methods

Study design and inclusion criteria

We performed a retrospective study of all inpatients discharged from our institution between January 1, 2016 and December 31, 2019. Our facility is a tertiary level 1 trauma center that serves Southern New Jersey. Almost one-third of our patients are transferred from other hospitals. The rest are directly admitted from outpatient sites or through the emergency department.

Patients > 18 years old under inpatient status were included for analysis. If a patient was originally admitted under observation status but then changed to inpatient, they were included in the study.

We excluded patients who expired, were transferred to another acute care facility, or left against medical advice, as well as those for whom readmission was already planned (such as cancer patients). We also excluded pregnant patients since their LOS is almost always consistent (48 or 72 hours depending on the type of delivery) and those with primary psychiatric diagnoses since placement into psychiatric facilities is largely due to non-medical factors. We also excluded patients that had missing values for LOS.

Outcomes and definitions

We recorded the following variables from the administrative database: age, gender, ethnicity, LOS, diagnosis-related group (DRG) type, case mix index (CMI), 30-day readmission, discharge destination, payor, discharging team, and substance abuse history.

We analyzed the relation between LOS and 30-day readmission in our patient population. The LOS was calculated by day of discharge, minus day of admission (presence of admission order). For the outcome readmission, we looked at the inpatient readmissions within 30 days to our institution. Readmissions classified as observations were not included for analysis. We used the CMI as a proxy for severity of illness [12].

The discharge destination was recorded as follows: home, skilled nursing facility (SNF)/rehab, and others. The “others” category for discharge includes prison, jail, inpatient hospice, and other facilities not specified. The discharging teams were grouped as follows: hospitalist, surgical, and others. The “others” category encompassed patients on services, including neurology, obstetrics and gynecology, maternal-fetal medicine, psychiatry, anesthesia, cardiology, critical care, infectious disease, and pulmonary. The DRG type was categorized as either medical or surgical. For the age variable, we considered all patients age 89 or older as being 89 years.

Statistical analysis

Categorical data are presented as percentages; continuous data are presented as mean (standard deviation). The population was subdivided between readmitted and non-readmitted patients. We used Chi-square analysis, Mann-Whitney U test, one-way analysis of variance (ANOVA), and t-test to assess for differences between the variables as appropriate.

We used a multivariate logistic regression model to assess for the independent association of LOS and readmission. We entered the following variables in our model: age, gender, race, year of discharge, discharge team, discharge diagnosis (medical vs surgical DRG), CMI, discharge destination (SNF/rehab vs not SNF/rehab), and insurance status (Medicare, Medicaid, private insurance, or other). The variables of LOS, age, and CMI were entered as continuous and all the others as categorical. We used a forward conditional model and variables were considered significant if p < 0.05. Data were analyzed using the Statistical Package for Social Sciences (SPSS), version 24.0 (IBM SPSS Statistics, Armonk, NY).

## Results

Patients and demographics

There were 105,339 patients discharged from our institution during the time period of the study. Of those, 13,366 patients did not have a readmission score calculated, 2,276 expired, 2,689 left against medical advice (AMA), 447 were discharged to hospice, 52 were discharged with intent to readmit, 752 were transferred to another facility, 2,137 patients had a primary psychiatric diagnosis, 2,470 were pregnancy-related, and 2,543 were cancer-related. An additional 250 patients did not have a LOS recorded. After excluding all those patients, 91,723 were included in the study. There were 22,531 patients discharged in 2016, 23,604 in 2017, 22,891 in 2018, and 22,697 in 2019. Readmitted patients over the years were 2,789 (2016), 2,803 (2017), 2,391 (2018), and 2,615 (2019).

Demographic variables are stratified by readmitted vs non-readmitted patients (Table 1). There were significant differences in age, gender, ethnicity, medical vs surgical, insurance status, and discharge destination. Male patients had higher rates of readmission (12.3 % vs 10.9%, p < 0.001). Caucasian patients had a 10.9% readmission rate as compared to a 14% rate for African Americans (p < 0.001). Readmission rates were higher for patients on hospitalist services (16.1%) versus those on surgical services (8.4%, p < 0.001). Readmission rates for patients with a primary medical diagnosis (13.2%) were higher versus a surgical diagnosis (9.2%, p < 0.001). Readmission rates for patients who were discharged to a facility were higher (14%) than those who were discharged to home (11.2%, p < 0.001). Insurance status were also associated with various readmission rates, ranging from readmission with Medicare patients (13.7%) as compared to private insurance (8.9%, p < 0.001).

**Table 1:** Demographic Variables by Readmission Status Rehab/SNF: rehabilitation center/skilled nursing facility

	All patients	Readmitted	Non-Readmitted	P-Value
Number Patients	91,723	10,598 (11.6%)	81,125 (88.4%)	
Age	55.9 (18.9)	57.8 (17.2)	55.7 (19.1)	P < 0.001
Female Gender/Male Gender	50,915/40,807	5,559 (10.9%)/5,039 (12.3%)	45,356 (89.1%)/35,768 (87.7%)	P < 0.001
Case Mix-Index	1.9 (1.83)	1.99 (1.89)	1.87 (1.82)	P < 0.001
Discharge year				
2016	22,531	2,789 (12.4%)	19,742 (87.6%)	P < 0.001
2017	23,604	2,803 (11.9%)	20,801 (88.1%)	
2018	22,891	2,391 (10.4%)	20,500 (89.6%)	
2019	22,697	2,615 (11.5%)	20,082 (88.5%)	
Race				
African American	22,658	3,172 (14%)	19,486 (86%)	P < 0.001
White	49,186	5,340 (10.9%)	43,846 (89.1%)	
Hispanic	14,371	1,705 (11.9%)	12,666 (88.1%)	
Other or Unable to Obtain	5,508	381 (6.9%)	5,127 (93.1%)	
Service				
Hospitalists	37,846	6,101 (16.1%)	31,745 (83.9%)	P < 0.001
Surgery	26,234	2,204 (8.4%)	24,030 (91.6%)	
Other	27,643	2,293 (8.3%)	25,350 (91.7%)	
Discharge diagnoses				
Medical	53,294	7,059 (13.2%)	46,235 (86.8%)	P < 0.001
Surgical	38,427	3,539 (9.2%)	34,888 (90.8%)	
Insurance status				
Medicare	37,317	5,103 (13.7%)	32,214 (86.3%)	P < 0.001
Medicaid	21,306	2,537 (11.9%)	18,769 (88.1%)	
Insurance	28,3286	2,509 (8.9%)	25,777 (91.1%)	
Other	4,814	449 (9.3%)	4,365 (90.7%)	
Discharge Destination				
Discharge Home	72,799	8,142 (11.2%)	64,657 (88.8%)	P < 0.001
Discharge Rehab/SNF	17,007	2,384 (14%)	14,623 (86%)	
Other Discharge	1,904	69 (3.6%)	1,835 (96.4%)	

Length of stay and readmission outcomes - univariate analysis

We then looked at differences in LOS between groups. The results are presented in Table 2. We analyzed differences for the overall population, by DRG (medical vs surgical), and discharge destinations. In all groups, readmitted patients had a significantly higher LOS.

**Table 2:** LOS by Readmission Status DRG: diagnosis-related groups; LOS: length of stay; Rehab/SNF: rehabilitation center/skilled nursing facility; SD: standard deviation

Geometric LOS	Total (days/SD)	Readmitted (days/SD)	Non-readmitted (days/SD)	P-value
All patients	5.37 (7.1)	6.87 (8.21)	5.18 (6.9)	P < 0.001
Medical DRG	4.46 (5.42)	5.3 (5.67)	4.33 (5.37)	P < 0.001
Surgical DRG	6.64 (8.7)	10 (11.08)	6.3 (8.35)	P < 0.001
Discharge home	4.21 (4.94)	5.45 (6.12)	4.06 (4.75)	P < 0.001
Discharged Rehab/SNF	9.89 (11.12)	11.6 (11.67)	9.61 (11)	P < 0.001

Regression analyses

We performed two multivariate regression analyses. First, for the outcome readmission, we included the geometric LOS as a continuous variable. Then, we ran another analysis of the outcomes of prolonged LOS. We defined prolonged LOS as LOS > median for the group (5.37) (Table 1). After running both regression analyses, we identified five factors that increased both readmission and prolonged LOS with significant odds ratios (Table 3).

**Table 3:** Multivariate Regression Analysis for Outcomes of Interest AA: African American; CI: confidence interval; CMI: case-mix index; DRG: diagnosis-related group; LOS: length of stay; Medicaid: Medicaid insurance; OR:  odds ratio

	Readmission (OR, 95% CI)	Prolonged LOS (OR, 95% CI)
LOS	1.02 (1.02 - 1.02)	
AA Race	1.24 (1.18 - 1.3)	1.11 (1.07 - 1.16)
CMI	1.05 (1.05 - 1.06)	2 (1.97 - 2.03)
Medical DRG	1.37 (1.30 - 1.15)	1.31 (1.26 - 1.37)
Medicaid	1.26 (1.18 - 1.48)	1.16 (1.11 - 1.21)
Hospitalist team	1.66 (1.56 - 1.76)	1.8 (1.73 - 1.89)

## Discussion

We found that patients with a longer LOS generally had a higher readmission rate. Traditionally, the concern has been that healthcare systems are incentivized to discharge patients early and this may lead patients to be discharged prematurely. There have been some studies, such as in congestive heart failure (CHF), suggesting that premature discharge may predispose to readmission [7-8]. Our study, however, suggests that shorter admissions do not necessarily translate into a greater risk for readmission. This suggests that more complicated patients, either medically or socially, require longer hospital stays, a supposition bolstered by the finding of a higher CMI among readmitted patients and that these more complex patients are also at higher risk for readmission. This potentially has profound implications in that it suggests the existing paradigm might not serve patients or health systems by incentivizing shorter lengths of stay in more complex patients. The substrate of these medically or socially complex patients may thus not obey a formulaic approach to the length of stay; their underlying conditions rather than delays and inefficiencies in health systems may be the factor that dictates the length of stay.

Patients with a primary medical diagnosis (13.2%) were at higher risk for readmission than those with a surgical diagnosis (11.2%). The group “surgical diagnosis” was inclusive of all surgeries and did not stratify elective procedures, such as hip replacements, from urgent cardiac surgeries or trauma. This catchment thus included patients who were presumably medically stable, had undergone preoperative testing, and had been risk-stratified and were deemed healthy enough to undergo surgery. The medical group lacked this healthier segment of the population, particularly since, by the definitions of the study, patients under observation status were excluded. Those patients on a hospitalist service similarly were at higher risk for readmission in the multivariate analysis (OR 1.8), likely for similar reasons of being more medically and/or socially complex related to their surgical counterparts. Complexity, as reflected in the CMI, helped to account for a portion of this effect (OR 1.05) but left the door open to the exploration of the myriad other factors contributing to higher readmission risk in this large population of patients. The association of readmission with Medicaid coverage (OR 1.26) may begin to get at some of these nuances as this may include patients with lower incomes.

Readmission rates declined from 2016 to 2019, while the absolute percentages appeared to show little variation (10.4% - 12.4%) (Table 1). Taken together then, readmission rates showed gradual improvement after the implementation of multidimensional interventions. There was an emphasis on ambulatory operations throughout this time and on easier access to outpatient appointments, especially within seven days of discharge. Inpatients were encouraged to create a log on their own electronic medical record. The hospital was generally very focused on safe transitions of care and had support staff to help navigate discharges. Patient safety initiatives focused on safe discharges also increased, but there was no one initiative that explained the improvement in readmissions over the four year time period, particularly as acuity gradually increased over the same time period (from 1.75 in 2016 to 1.88 in 2019).

LOS was found to be consistently higher in readmitted patients across all subgroups. The LOS was significantly higher in surgical patients as compared to medical patients. This discrepancy may be explained by the fact that surgical patients may require optimization prior to surgery, may have complicated conditions requiring longer hospital stays, and may have challenging social and economic backgrounds.

Assessment of demographic characteristics revealed discrepancies in the readmission rates for African Americans (14%) and Hispanics (11.9%) as compared to Caucasians (10.9%). African American race patients were also found to have a higher LOS. These results are consistent with findings from other studies [13-15]. This may reflect differences in access to care and socioeconomic status and points to a higher risk population for which additional safeguards should be considered to maintain a safe discharge. Chart review to explore this phenomenon was not possible by the nature of this study design but should be further studied in a more detailed, individual patient analysis.

Limitations to our study include that it is provided only a single center perspective which reflects the experience at a large, tertiary care center. We derived our data from a large database. Large database studies provide an excellent broad-based perspective to the nature of a problem but do not allow for a deep dive into specific questions. As such, we can only make suppositions into the underlying forces governing the effects we report. However, it does make intuitive sense that patients with longer LOS or those on medical services are sicker or more complex and, therefore, at higher risk for readmission rather than that the longer LOS’s represent delays on the part of the physician or healthcare system. The de-identified data available to us did not permit exploration of the readmission diagnosis on individual patients to be able to say if the reason for readmission was the same as the original admission.

Regardless of the readmission diagnosis, however, it may be reasonable to advocate for change in reimbursement based on LOS and to switch to a model based on the severity of illness and medical and biopsychosocial complexity. Such a focus would not penalize health systems with sicker patients and would distribute resources in a more equitable fashion. This should not distract from goals for increased healthcare efficiency and minimization of logistical burdens but rather should be seen to bolster the individualized care of patients, foster resiliency of health systems, and represent a more equitable distribution of resources. 

## Conclusions

Contrary to our initial hypothesis, we found that general medical patients with higher LOS had a higher likelihood of being readmitted to the hospital after adjusting for other variables. It is possible that factors not captured in the current dataset may help explain both the increase in LOS and readmission risk. Future studies should analyze the individual factors that result in higher rates of readmissions and prolonged length of stay.
